# Facial profile changes due to bone cement graft to manage the hyperactive muscles of the gingival smile

**DOI:** 10.1590/2177-6709.25.2.044-051.oar

**Published:** 2020

**Authors:** Érica Miranda de Torres, José Valladares-Neto, Karina de Oliveira Bernades, Luis Fernando Naldi, Hianne Miranda de Torres, Alexandre Leite Carvalho, Carlos Estrela

**Affiliations:** 1 Universidade Federal de Goiás, Faculdade de Odontologia, Divisão de Reabilitação Oral (Goiânia/GO, Brazil).; 2 Universidade Federal de Goiás, Faculdade de Odontologia, Divisão de Ortodontia (Goiânia/GO, Brazil).; 3 Universidade Federal de Goiás, Programa de Pós-graduação em Odontologia, Faculdade de Odontologia (Goiânia/GO, Brazil); 4 Universidade Federal de Goiás, Faculdade de Odontologia, Divisão de Periodontia (Goiânia/GO, Brazil).; 5 Universidade Federal de Goiás, Faculdade de Odontologia, Divisão de Endodontia (Goiânia/GO, Brazil).

**Keywords:** Gingival smile, Bone cement, Polymethyl methacrylate, Facial profile

## Abstract

**Objective:**

To evaluate facial profile changes promoted by polymethyl methacrylate (PMMA) cement graft to reduce excessive gingival display due to hyperactivity of the elevator muscles of the upper lip during smiling.

**Methods:**

Eleven patients (all females, age range: 20 to 43 years) presenting gingival smile that were treated with PMMA cement grafts in a private clinic were selected for this retrospective study. Three angular and ten linear cephalometric facial profile measurements were performed preoperatively (baseline, T_1_) and at least 6 months postoperatively (T_2_). Differences between T_1_ and T_2_ were verified by Wilcoxon test, and the correlation between the thickness of the graft and facial profile changes was statistically evaluated by Spearman’s Coefficient test. The significance level was set at *p*< 0.05.

**Results:**

The nasolabial angle (*p*= 0.03) and the labial component of the nasolabial angle showed statistically significant differences (*p*= 0.04), with higher values in T_2_. No correlations were found between the graft thickness and the statistically significant facial profile changes (*p*> 0.05).

**Conclusions:**

The PMMA bone cement graft projected the upper lip forward, thereby increasing the nasolabial angle without affecting the nasal component. No correlations between the graft thickness and the facial profile changes were detected.

## RESUMO

**Objetivo:**

Avaliar as alterações do perfil facial promovidas pelo enxerto de cimento de polimetilmetacrilato (PMMA) para redução da exposição gengival excessiva devida à hiperatividade dos músculos elevadores do lábio superior durante o sorriso.

**Métodos:**

Onze pacientes (todos do sexo feminino, faixa etária de 20 a 43 anos) com sorriso gengival, tratados com enxerto de cimento de PMMA em clínica privada, foram selecionados para este estudo retrospectivo. Três medidas cefalométricas angulares e dez lineares do perfil facial foram realizadas no pré-operatório (T_1_) e com pelo menos seis meses de pós-operatório (T_2_). As diferenças entre T_1_ e T_2_ foram verificadas pelo teste de Wilcoxon, e a correlação entre a espessura do enxerto e as alterações do perfil facial foi avaliada estatisticamente pelo Coeficiente de Spearman. O nível de significância foi estabelecido em *p*< 0,05.

**Resultados:**

o ângulo nasolabial (*p*= 0,03) e o componente labial do ângulo nasolabial apresentaram diferenças estatisticamente significativas (*p*= 0,04), com maiores valores em T_2_. Não foram encontradas correlações estatisticamente significativas (*p*> 0,05) entre a espessura do enxerto e as alterações do perfil facial.

**Conclusões:**

O enxerto de cimento ósseo de PMMA projetou discretamente o lábio superior, aumentando o ângulo nasolabial sem afetar o componente nasal. Não foram detectadas correlações entre a espessura do enxerto e as alterações do perfil facial.

## INTRODUCTION

The smile is the spontaneous expression linked to joy, pleasure and receptivity.^1^ Tarantili et al.^2^ defined a pleasant smile as one in which there is complete exposure of the anterior maxillary teeth and a mild gingival display of 1 to 3 mm. An excessive gingival display greater than 3 mm is considered unpleasant or unattractive, and is popularly called “gummy smile”.^1^ ^,^ ^3^ ^,^ ^4^

Several etiological factors have been associated to gingival smile, and it is important for the clinician to properly identify its etiology, for an adequate treatment. These factors occur separately or in combination,^4^ ^-^ ^8^ and according to the origin, they can be grouped into: dental (excessive dentoalveolar extrusion^9^), gingival (altered passive eruption^10^ ^,^ ^11^ or gingival enlargement^12^), skeletal (excessive maxillary vertical growth^8^) or muscular (short upper lip or hyperactivity of the elevator muscles of the upper lip^1^ ^,^ ^2^ ^,^ ^6^). For this reason, various treatments have been proposed according to the etiology of the gingival smile, including orthodontic intrusion,^5^ ^,^ ^7^ ^,^ ^9^ gingivectomy,^7^ ^,^ ^13^ periodontal plastic surgery,^13^ maxillary teeth intrusion by skeletal anchorage,^14^ ^,^ ^15^ maxillary impaction by orthognathic surgery;^7^ ^,^ ^8^ upper lip repositioning,^16^ ^-^ ^19^ and botulinum toxin injection.^20^ ^-^ ^22^

**Figure 1:**
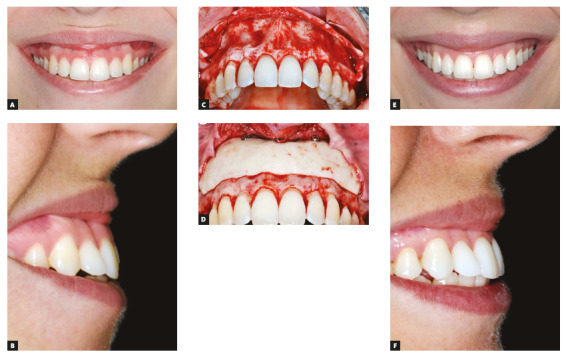
Pre-surgical photographs (A and B); Trans-surgical photographs, showing before (C) and after (D) fixation of bone cement graft on subnasal depression, associated with periodontal plastic surgery; Post-surgical outcomes (E and F).

Recently, a new surgical technique for the management of the gingival smile was proposed by Naldi et al.^23^ ^-^ ^25^ The technique consists in implanting a bone cement graft based on polymethyl methacrylate (PMMA) in the anterior maxilla below the pyriform aperture. According to the authors, some patients with a gingival smile have a major subnasal depression that allows the upper lip to be lodged during spontaneous smile. The PMMA bone cement graft fills this depression, preventing excessive displacement of the upper lip during contraction. The graft, associated with esthetic crown lengthening, has been shown to be effective in reducing gingival display.^23-25^ An internal bevel incision followed by a reflected full-thickness flap is performed until exposure of the subnasal depression. The PMMA bone cement is indicated by the manufacturer for use in fixing orthopedic prostheses to bone tissue. It is manipulated (powder and liquid) and adapted to the region of interest. After polymerization, the PMMA graft is fitted with drills, for better volume and conformation. Two screw fixations are used to immobilize the PMMA graft in the subnasal depression. Sutures are inserted and removed 10 days later.^23-25^ Figure 1 illustrates this surgical technique and the outcomes.

PMMA is biocompatible and effective for reduction of the gingival smile; in addition, complications or adverse reactions, such as implant or bone resorption in the recipient region, were not reported in the medium term.^23-25^ However, there is still no information about the impact of this technique on the changes in facial profile. Thus, the aim of the present study was to test the null hypothesis that PMMA cement implanted to manage the gingival smile is not able to introduce changes in facial profile at rest. In addition, this study also aimed to determine whether a correlation exists between the thickness of the bone cement and a possible change in facial profile.

## MATERIAL AND METHODS

The research protocol of this retrospective study was reviewed and approved by the Research Ethics Committee of the Federal University of Goiás (Goiânia, Brazil; CEP/UFG - 065/2011). The STROBE guidelines for observational studies were followed.^26^

### Sample

The sample initially comprised 38 patients submitted to the implantation of bone cement based on PMMA (Aminofix 3, Groupe Lepine, France) at a private clinic in the city of Goiânia, Goiás, Brazil. The same surgeon operated all the patients, between November 2007 and April 2012. Among the initial sample, 11 met the inclusion criteria. All were women, aged from 20 to 43 years. The inclusion criteria were as follows: patients with gingival smile due to hyperactive muscles, treated with bone cement graft; 18 years-old or older; presence of pre- and postsurgical cephalometric radiographs with relaxed lips. The cases of hyperactivity of the elevator muscle of the upper lip involved both functional and morphological evaluation. Firstly, patients presented with high lip dynamics and, secondly, with a major subnasal depression that allows the upper lip to lodge during a spontaneous smile. The exclusion criteria were: poor image quality; cleft lip or cleft lip and palate; craniofacial syndromes; and other surgical interventions that could change the soft and hard tissues of the face during the period of study. For this study, cephalometric radiographs had been taken prior to treatment (T_1_) and at least six months after surgery (T_2_) from 11 patients submitted to surgical implantation of grafts based on PMMA.

### Cephalometric analysis

The cephalometric radiographs (lateral cephalometric radiographs) were taken using the same cephalostat at T_1_ and T_2_ with an enlargement factor near to 1.0645 (6.45 percent). The patients were in a standing position and adequately protected, with the teeth in centric occlusion, lips in relaxed position, and Frankfurt plane parallel to the floor. Radiographic films were developed in an automatic processor (Flat Co., Japan).

**Figure 2:**
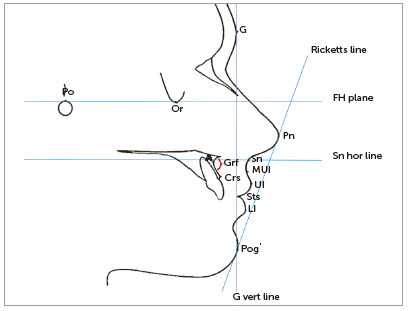
Definitions of cephalometric landmarks and reference planes used in the study.

Facial profile was outlined by hand, on acetate paper in a darkened room, by the same experienced and calibrated investigator. A prior calibration was performed with three cephalometric radiographs traced twice by the same investigator, with an interval of two weeks. After confirmation of excellent concordance (ICC > 0.90), the overall sample was measured. The cephalometric tracing was composed by the anterior contour of the maxilla, symphysis, maxillary central incisors, external auditory canal and lower border of the orbit, besides the soft tissue profile (Fig 2, Tab. 1). Thirteen skeletal and soft tissue cephalometric landmarks, being three angular and ten linear variables, were measured, including: the nasolabial angle, the nasal component of the nasolabial angle, the labial component of the nasolabial angle, upper lip concavity, upper lip length, upper lip vermilion length, upper lip thickness, subnasal thickness, upper lip protrusion, upper lip anteroposterior position, lower lip anteroposterior position, and graft thickness (Tab. 2). 

**Table 1:** Definitions of cephalometric landmarks and reference planes.

Po (Porion)	The upper most point of the body of the external auditory meatus, usually regarded as coincidental with the ear rods of the cephalostat
Or (Orbitale)	The lowest point on the infraorbital margin
G (Glabela)	The most prominent point on the glabela
Pn (Pronasal)	Most prominent point of the nose in the soft profile
Sn (Subnasal)	Point located at the intersection of the upper lip and base of the nose
Ul (Upper lip)	Point located in the most anterior region of the red portion of the upper lip
MUl (Medium Upper Lip)	Midpoint between points Ul and Sn
Sts (Stomion)	Highest midline point of upper lip
Ll (Lower lip)	Point located in the most anterior region of the red portion of the lower lip
Pog’ (Soft Pogonion)	Most anterior point of the outline of the soft chin
Crs (Upper bone crest)	Interproximal bone crest of the upper central incisors
A (Subspinale - A point)	The deepest midline point between the anterior nasal spine and the prosthion
Grf (Graft)	Most anterior point of the graft of PMMA
Ricketts line (Aesthetic line of Ricketts)	Line drawn from the tip of the nose (Pn) to the tip of the chin (Pog’)
FH plane (Frankfurt horizontal plane)	Connecting Po and Or points
Sn hor line	Horizontal line parallel to the FH plane passing through Sn point
G vert line	Vertical line perpendicular to the FH plane passing through G point

**Table 2:** Linear and angular cephalometric measurements used the study.

Name	Measure	Description
Nasolabial angle	Pn.Sn.Ul (degrees)	The angle formed by Pn, Sn and Ul points
Nasal component of the nasolabial angle	(Pn-Sn).Sn hor (degrees)	The angle between nasal base (Pn-Sn) and Sn hor line
Labial component of the nasolabial angle	(Sn-Ul).Sn hor (degrees)	The angle between the upper lip length (Sn-Ul) and Sn hor line
Upper lip concavity	MUl-(Sn-Ul) (mm)	The minor linear distance from point MUl to Sn-Ul line
Upper lip length	Sn-Ul (mm)	The linear distance from points Sn and Ul
Upper lip vermilion length	Ul-Sts (mm)	The linear distance from points Ul and Sts
Upper lip thickness	Crs-Ul (mm)	The linear distance between points Crs and Ul
Subnasal thickness	A-Sn (mm)	The linear distance between points A and Sn
Medium upper lip thickness	A-MUl (mm)	The linear distance between points A and MUl
Upper lip protrusion	Gvert-Ul (mm)	The linear distance between point Ul and G vert line
Upper lip anteroposterior position	Ul-(Pn-Pog’) (mm)	The minor linear distance between point Ul and esthetic line of Ricketts (Pn-Pog’)
Lower lip anteroposterior position	Ll-(Pn-Pog’) (mm)	The minor linear distance between point Ll and esthetic line of Ricketts (Pn-Pog’)
Graft thickness	A-Grf (mm)	The linear distance between points A and Grf

### Method error

To determine the reliability of the cephalometric method, all 11 cephalometric radiographs from T_1_ and T_2_ were traced and measured twice, with a one-month interval, by the same investigator. Systematic errors were evaluated with the intraclass correlation coefficient (ICC). Excellent concordance between the two measurements at T_1_ and T_2_ was found (average: 0.903, range 0.639 - 0.974), resulting in no systematic errors.

### Statistical analysis and power calculation

The preliminary assessment of data at T_1_, T_2_, and T_1_-T_2_ changes revealed the presence of both nonnormal distribution (Kolmogorov-Smirnov test) and no homogeneity of variances (Levene’s test) for all the variables. T_1_ and T_2_ variables were compared with the Wilcoxon signed-rank test. Correlations between possible statistically significant differences at T_1_, T_2_ and T_1_-T_2_ changes and the thickness of the graft (A-Grf) were verified with Spearman’s Coefficient test. The significance level was set at *p*< 0.05. SPSS 17.0 for Windows (SPSS Inc., Chicago IL, USA) was used for the statistical analysis. The power of the study was based on the sample size available and measurements that present statistically significant differences.

## RESULTS

**Figure 3:**
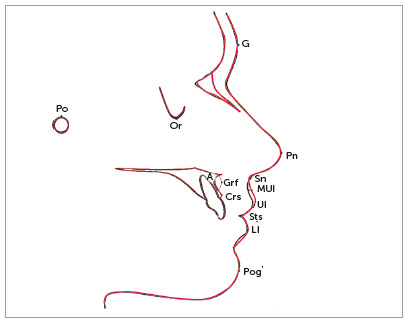
Superimposed cephalometric tracing before (T1, black) and after (T2, red) treatment (superimposed on the Sella-Nasion line centered on Sella).

The median, minimum, and maximum values ​​of the cephalometric measurements are described in Table 3. Statistically significant differences between T_1_ and T_2_ angular and linear measurements were found for the nasolabial angle (Pn.Sn.Ul) (*p*= 0.03) and the labial component of the nasolabial angle [(Sn-Ul).Sn hor] (*p*= 0.04). The overall changes can be summarized by a representative superimposed cephalometric tracings before (T_1_) and after (T_2_) surgical treatment (Fig. 3). 

**Table 3:** Descriptive statistics and p values for comparisons between T1 and T2.

Measurement	Time	Median	Q25-Q75	P value
Pn.Sn.Ul (degrees)	T1	111	109-123	0.03*
	T2	116	111.5-129	
(Pn-Sn).Sn hor (degrees)	T1	33	30.5-35	0.475
	T2	33.5	33-36.5	
(Sn-Ul).Sn hor (degrees)	T1	79	75-88	0.04*
	T2	82	78.5-88.5	
MUl-(Sn-Ul) (mm)	T1	-2	-3-(-2)	0.618
	T2	-2	-3-(-1.5)	
Sn-Ul (mm)	T1	17	16-20	0.75
	T2	19	15-21	
Ul-Sts (mm)	T1	10.5	9.5-11	0.38
	T2	10.5	9.5-11	
Crs-Ul (mm)	T1	15	14-17	0.326
	T2	14	12-17	
A-Sn (mm)	T1	16	14.5-17.5	0.55
	T2	16	14-19	
A-MUl (mm)	T1	16	14-17	0.392
	T2	15	14-17.5	
Gvert-Ul (mm)	T1	6.5	6-10	0.15
	T2	7	6-9	
Ul-(Pn-Pog’) (mm)	T1	-3.5	-5-(-1)	0.119
	T2	-2.5	-5.5-(-1)	
Ll-(Pn-Pog’) (mm)	T1	-2	-2.5-2	0.337
	T2	-0.5	-2-2	

* Statistical significant differences based on Wilcoxon’s test (p < 0.05)

The thickness of the graft (A-Grf) ranged from 4 to 7 mm. Correlation analysis was performed to determine whether there was a correlation between the thickness of the graft (A-Grf) and facial profile changes. The correlation test was performed only for the measurements that were statistically significant. T_2_ measurements were used when it was possible to obtain A-Grf, and differences between T_1_ and T_2_ (T_1_- T_2_). Spearman’s Coefficient test showed no statistically significant correlations (Tab. 4).

**Table 4:** Correlation tests between A-Grf and measurements that were statistical significant.

Correlation tested	Spearman’s rho (r)	P value
Pn.Sn.Ul (degrees) and A-Grf	-0.103	0.763
Dif Pn.Sn.Ul (degrees) and A-Grf	0.3	0.370
(Sn-Ul).Sn hor (degrees) and A-Grf	-0.185	0.586
Dif (Sn-Ul).Sn hor (degrees) and A-Grf	0.141	0.679

Dif is the difference between T1 and T2 (T1-T2) for the indicated measurement.

The calculation of the test power was based on the average and standard deviation of the nasolabial angle (Pn.Sn.Ul) and the labial component of the nasolabial angle [(Sn-Ul).Sn hor]. Considering an alpha level of 5%, the sample size (n = 11), and a clinically meaningful difference of 10 degrees to the nasolabial angle, and 8% for the labial component of the nasolabial angle, the power calculation was 0.83 and 0.87, respectively.^27^

## DISCUSSION

Many techniques have been developed for treating gingival smiles. However, there is a lack of studies related to cephalometric changes provided by these techniques. Therefore, this original study investigated the cephalometric changes that occurred due to the use of bone cement based on PMMA. The null hypothesis was rejected, since cephalometric alterations in the nasolabial angle and the labial component of the nasolabial angle were observed with the use of the bone cement based on PMMA. All patients who were available and met the inclusion criteria were evaluated. The power calculation indicated adequate power for statistical inferences.

A gingival smile is reported as being unattractive by dentists and lay people.^4^ It is primarily an aesthetic discomfort rather than a pathology, and treatment depends on the perception and willingness of the patient. A gingival smile is multifactorial and different approaches can be indicated based on its etiology.^5^ ^,^ ^7^ Hyperactivity of the upper lip can be identified by contraction of the elevator muscles of the upper lip, leaving it at 30% of its original height at rest.^1^ This means 20% to 25% more muscular capacity to raise the upper lip than in subjects without a gingival smile.^1^ ^,^ ^6^ Some approaches have been recommended for this etiology, including upper lip repositioning,^16^ ^-^ ^19^ botulinum toxin injection,^20^ ^-^ ^22^ and PMMA bone cement graft.^23^ ^-^ ^25^

The PMMA bone cement is marketed in Brazil and registered at the National Sanitary Surveillance Agency (ANVISA) valid until 2028. Its preliminary indication was intended for orthopedic medical surgeries. In the present study, a PMMA graft was proposed to fill the major subnasal depression observed in some patients with gingival smile. The graft was able to fill this depression, resulting in reduced gingival display. It was hypothesized that this depression contributes to the lack of lip support and, when smiling, the lip tends to contract, shorten, and become lodged in this depression.^23^ ^-^ ^25^ Surgery with graft-based PMMA bone cement would probably interfere in the dynamics of the smile by limiting the retraction of elevator muscles.^23-25^ It is not clear how much of this reduction in gingival display may be due to the PMMA graft, since the graft has been associated with aesthetic crown lengthening.

However, it should be borne in mind that the technique has been shown to be effective in reducing the gingival smile. Clinical reports have demonstrated the stability and biocompatibility of the PMMA graft in the medium term (12, 18, and 22 months).^23^ ^-^ ^25^ But could the implantation of PMMA bone cement promote facial profile changes? There are no scientific studies to clarify these assumptions.

In Medicine, PMMA bone cement grafts have been used in cranioplasty, with high stability and a low complication rate.^28^ ^,^ ^29^ PMMA grafts are biocompatible,^30^ very stable, and inert over time, and are considered a cost-effective and safe technique.^28^ ^,^ ^29^ In Dentistry, PMMA has also been used in the maxillofacial area for tumors, trauma, and TMJ ankylosis.^31^

The pioneering objective of this study was to evaluate the impact of PMMA bone cement grafts to manage the gingival smile in facial profile at rest, thereby enabling the understanding of some of the possible side effects of this intervention. The hypothesis that a PMMA graft was able to introduce cephalometric changes in facial profile was accepted.

The cephalometric results of this study demonstrated that the graft does not cause many significant changes in the facial profile. However, a small protrusion of the upper lip, promoting a slight increase of the nasolabial angle, without affecting the nasal component, was verified. It is important to highlight as a limitation of this study that the cephalometric method is a static and lateral evaluation. Frontal and functional evaluations still require investigation.

Cephalometric analysis has been conducted to evaluate facial changes due to other surgical procedures. Maxillary impaction changed the vertical and anteroposterior maxilla position, leading to nasal protraction, upper lip retraction due to retraction of the maxillary incisors, and soft tissue movement of the chin.^32^ Compared to maxillary impaction, a PMMA bone cement graft can be advantageous, since it does not seem to induce major changes in facial physiognomy, and have a low risk of neurosensory deficit and bleeding, as described for orthognathic surgery.^33^ However, the PMMA graft may not be the treatment of choice for resolving cases of gingival smile whose etiology is excessive vertical growth of the maxilla with greater severity.^23^

Compared to other treatment options to manage hyperactivity of the upper lip, it is important to point out that the effect of PMMA graft is not temporary, as has been described for the approach with injection of botulinum toxin.^20^ ^-^ ^22^ As regards upper lip repositioning, it is difficult to discuss and try to compare it with PMMA graft effects, because the approaches are very distinct.^16^ ^-^ ^19^

There was no correlation between the PMMA graft thickness and the facial profile changes. This may be explained by the small variability in the thickness of the grafts, which ranged from 4 to 7 mm. Even so, results of this study indicate a slight and long-term change in facial profile with PMMA bone cement. The PMMA graft added volume to the upper lip, projecting it forward. This facial profile change may be considered unpleasant for some patients, and therefore, this should be considered for the indication of the technique. Studies regarding patients’ perceptions about the treatment of gingival smile with PMMA grafts and to clarify many other aspects involved in the technique are necessary.

### Limitations

This was a retrospective study with the main sample selected from clinical files. The selection of the sample was possible due to the availability of records of cases treated by means of the surgical procedure under analysis. No control group was used due to ethical reasons, and the comparison was performed between before and after the surgical procedure. Statistical difference found only for the nasolabial angle may be due to the fact that the sample was too small and unable to reveal the actual results related to type II error. However, the high power obtained for this inference makes it reliable. The 2-D cephalometric measurements confirmed good reproducibility, and a 3-D evaluation was not the scope of this study. Moreover, the decision to submit these patients to higher X-ray exposures was not justified. The female sample limited the generalization of the data, however, there is no biological reason to find difference between the sexes.

## CONCLUSIONS

» The PMMA bone cement graft to manage the hyperactive musculature during gingival smile projected the upper lip forward about 1mm, increasing the nasolabial angle without affecting the nasal component.
» No correlations between the implant thickness and the facial profile changes was detected.
